# Record‐Breaking Increases in Arctic Solar Ultraviolet Radiation Caused by Exceptionally Large Ozone Depletion in 2020

**DOI:** 10.1029/2020GL090844

**Published:** 2020-12-10

**Authors:** Germar H. Bernhard, Vitali E. Fioletov, Jens‐Uwe Grooß, Iolanda Ialongo, Bjørn Johnsen, Kaisa Lakkala, Gloria L. Manney, Rolf Müller, Tove Svendby

**Affiliations:** ^1^ Biospherical Instruments Inc. San Diego CA USA; ^2^ Environment and Climate Change Canada Toronto Ontario Canada; ^3^ Forschungszentrum Jülich Jülich Germany; ^4^ Finnish Meteorological Institute Helsinki Finland; ^5^ Norwegian Radiation and Nuclear Safety Østerås Norway; ^6^ Finnish Meteorological Institute Sodankylä Finland; ^7^ NorthWest Research Associates Socorro NM USA; ^8^ New Mexico Institute of Mining and Technology Socorro NM USA; ^9^ NILU‐Norwegian Institute for Air Research Kjeller Norway

**Keywords:** solar UV radiation, total ozone column, UV Index, Arctic, anomaly

## Abstract

Measurements of solar ultraviolet radiation (UVR) performed between January and June 2020 at 10 Arctic and subarctic locations are compared with historical observations. Differences between 2020 and prior years are also assessed with total ozone column and UVR data from satellites. Erythemal (sunburning) UVR is quantified with the UV Index (UVI) derived from these measurements. UVI data show unprecedently large anomalies, occurring mostly between early March and mid‐April 2020. For several days, UVIs observed in 2020 exceeded measurements of previous years by up to 140%. Historical means were surpassed by more than six standard deviations at several locations in the Arctic. In northern Canada, the average UVI for March was about 75% larger than usual. UVIs in April 2020 were elevated on average by about 25% at all sites. However, absolute anomalies remained below 3.0 UVI units because the enhancements occurred during times when the solar elevation was still low.

## Key Points

- Record‐breaking low total ozone over the Arctic in March and April 2020 led to unprecedently large increases of UV radiation at Earth's surface
- Relative increases in the noontime UV Index exceeded 100% at several Arctic locations and surpassed historical means by more than six standard deviations
- Despite large relative increases in UV radiation, absolute increases remained small because enhancements occurred at low solar elevations

## 1 Introduction

Total ozone columns (TOC) over the northern polar cap (latitudes > 63°N) were exceptionally low in late winter and early spring (February–April) of 2020 (Lawrence et al., 2020). The average TOC in 2020 for this 3‐month period was 340 DU, which is 100 DU below the mean of measurements between 1979 and 2019 and the lowest value since the start of satellite measurements in 1979. The low TOCs in 2020 were partially caused by an exceptionally strong, cold, and persistent stratospheric polar vortex, which provided ideal conditions for chemical ozone destruction to occur (Grooß & Müller, 2020; Manney et al., 2020; Wohltmann et al., 2020). Temperatures low enough to promote polar stratospheric cloud formation within the vortex developed early in the season and enclosed about one third of the vortex volume on average. These conditions are unprecedented since at least 1979/1980; thus, 2019/2020 had the greatest Arctic ozone loss potential on record. The conditions leading to anomalously low TOCs are discussed further in other papers of this special collection (Grooß & Müller, 2020; Lawrence et al., 2020; Manney et al., 2020; Wohltmann et al., 2020).

Here we report on the effect of these extraordinarily low TOCs on erythemal (sunburning) UVR levels measured by ground‐based instruments at 10 Arctic and subarctic locations and observed by the Ozone Monitoring Instrument (OMI) onboard the Aura satellite of the National Aeronautics and Space Administration (NASA).

A low‐ozone event similar to that observed in 2020, also leading to substantial increases in erythemal UVR, occurred in 2011. Bernhard et al. (2013) showed that the noontime UV Index (UVI) exceeded the climatological mean by up to 77% in Alaska, Canada, and Greenland and by more than 150% in Scandinavia. The cumulative UV dose measured during the low‐ozone period between late March and early April 2011 exceeded the mean by over two standard deviations (SD) at 11 of 13 sites studied. Enhancements beyond three (four) SD were observed at seven (two) sites. As shown below, anomalies observed in 2020 exceeded those in 2011.

Our assessment is based on ground‐based and satellite UVR measurements. Ground‐based measurements are generally more accurate than satellite data (Bernhard et al., 2015). This is particularly the case for the Arctic because of the difficulty in distinguishing between clouds and snow from space. Algorithms that calculate UVR at the surface from observations by OMI (Lindfors et al., 2018; Tanskanen et al., 2006) rely on an albedo climatology (Tanskanen, 2004), which may have unrealistic values at some locations. This climatology also does not take year‐to‐year changes in albedo into account. When the albedo climatology exceeds the actual albedo, satellite data may be biased high by as much as 55%; conversely, when the climatology is too low, data can be biased low by up to 59% (Bernhard et al., 2015). Despite these limitations, satellite data are indispensable because of their near‐global spatial coverage. In contrast, Arctic ground‐based measurements are sparse and reliable measurements over vast areas (Alaska, Greenland, and Siberia) have either been discontinued or never been established.

## 2 Materials and Methods

### 2.1 Locations

Ground‐based data from 10 Arctic and subarctic locations were used in this analysis. Sorted by decreasing latitude, the 10 sites are Alert, Eureka, Ny‐Ålesund, Resolute, Andøya, Sodankylä, Trondheim, Finse, Østerås, and Churchill. Essential information about these sites is provided in Table 1, and their locations are indicated in Figure 2. Climatic conditions for all sites except Churchill were summarized by Bernhard et al. (2013). Churchill is located on Hudson Bay away from populated areas. The climate is subarctic with long, very cold winters and short, cool to mild summers. The shallow Hudson Bay freezes during winter, eliminating maritime moderation of the climate.

**Table 1:** Site Overview

Site	Alert	Eureka	Ny‐Ålesund	Resolute	Andøya
Country	Canada	Canada	Norway	Canada	Norway
Site acronym	ALT	EUR	NYA	RES	AND
Affiliation^a^	EC	EC	NILU/DSA	EC	NILU/DSA
Latitude (°)	82.5 N	79.99 N	78.92 N	74.72 N	69.28 N
Longitude (°)	62.32 W	85.93 W	11.92 E	94.98 W	16.01 E
Elevation (m)	220	635	45	26	380
Instrument	Brewer^b^	Brewer^c^	GUV‐541	Brewer^d^	GUV‐541
Period	1995–2020^e^	2001–2020	1996–2020	1991–2020	2000–2020
Data source^f^	WOUDC	WOUDC	NILU/DSA	WOUDC	NILU/DSA
Observations per hour	4 (median)	4 (median)	60	4 (median)	60
Uncertainty UVI (*k* = 2)^g^	6%	6%	6%	6%	6%
Site	Sodankylä	Trondheim	Finse	Østerås	Churchill
Country	Finland	Norway	Norway	Norway	Canada
Site ID	SOD	TRH	FIN	OST	CHU
Affiliation^a^	FMI	DSA/NILU	DSA/NILU	DSA/NILU	EC
Latitude (°)	67.37 N	63.42 N	60.60 N	59.95 N	58.74 N
Longitude (°)	26.63 E	10.40 E	7.52 E	10.60 E	94.07 W
Elevation (m)	179	65	1210	135	26
Instrument	Brewer^h^	GUV‐541	GUV‐541	GUV‐541	Brewer^i^
Range of years	1991–2020	1996–2020	2003‐2020^j^	1999‐2020	2000‐2020
Data source^f^	FMI	NILU/DSA	NILU/DSA	NILU/DSA	WOUDC
Observations per hour	2 or 3	60	60	60	4 (median)
Uncertainty UVI (*k* = 2)^g^	6%	6%	6%	6%	6%

^a^ NILU: Norwegian Institute for Air Research; DSA: Norwegian Radiation and Nuclear Safety Authority; EC: Environment and Climate Change Canada; FMI: Finnish Meteorological Institute.

^b^ 1995: Brewer MKII #012; 2000–2020: Brewer MKII #019 and Brewer MKV #029.

^c^ Brewer MKV #69.

^d^ 1991–2004, 2007, 2010: Brewer MKII #031; 2003, 2004, 2008–2012: Brewer MKII #013; 2013–2018: Brewer 100 MKIII #205; 2019–2020: Brewer MKIII #205 and Brewer MKII #31.

^e^ No data for 1996–1999.

^f^ See Data Availability Statement for links to data; WOUDC: World Ozone and UV Data Centre.

^g^ See Bernhard et al. (2013).

^h^ Brewer MKII #037.

^i^ 2000–2014: Brewer MKII #026; 2015–April 2019: Brewer MKIII #203; May 2019–July 2020: Brewer MKIII #239.

^j^ No data for June 2020.

### 2.2 Instruments, Measurement Protocols, Data Processing

Instruments used for ground‐based measurements are a subset of those described by Bernhard et al. (2013), plus Brewer spectrophotometers at Churchill. Measurement protocols and data processing methods are identical to those described by Bernhard et al. (2013). Brewer data from Churchill were processed in the same way as those from Alert, Eureka, and Resolute.

UVR is quantified with the UVI, which is a measure of the capacity of UVR to cause erythema (sunburn) in human skin (McKinlay & Diffey, 1987; WHO, 2002). In addition to its dependence on TOC, the UVI depends on solar elevation, clouds, surface albedo, and aerosols (Bais et al., 2019; Weatherhead et al., 2005). Historical ground‐based and satellite data (Bernhard et al., 2013, 2015) suggest that the UVI in the Arctic ranges from 0 to about 7, with the smallest annual peak radiation levels (UVI values < 4) observed at the northernmost sites. UVI values ≤ 5 indicate low to moderate risk of erythema (WHO, 2002).

UVI anomalies discussed below are based on UVIs averaged over 2‐hr periods centered at local solar noon (hereinafter “noontime measurements”). Additional results using the daily maximum UVI and the daily erythemal UV dose (i.e., erythemal irradiance integrated over 24 hr) are available as supporting information (SI).

The Norwegian sites provide UVI data in 1‐min intervals, while data from the other sites using spectroradiometers are available at rates ranging from 2–4 scans per hour (Table 1). The different sampling schemes have only a small effect on anomalies calculated from these data: For noontime measurements, the bias introduced by subsampling to 15 min is smaller than ±0.1% and the standard deviation of daily biases is smaller than ±4% (Bernhard et al., 2013). Data presented here from the Norwegian sites were subsampled to four measurements per hour to resemble the measurement frequency of the other instruments.

TOC data complementing UVI measurements from 2005 to 2020 were measured by OMI (Levelt et al., 2018). TOC data prior to 2005 are based on “overpass” data from Total Ozone Mapping Spectrometers (TOMS) onboard the Nimbus 7, Meteor 3, and Earth Probe satellites (see Data Availability Statement).

We used three metrics for evaluating TOC and UVI anomalies from ground‐based measurements: the relative difference between measurements in 2020 and the mean of measurements performed during one of four reference periods defined below, the absolute difference between 2020 and this mean, and the number of standard deviations by which measurements in 2020 exceeded this mean. The last metric was calculated by dividing the absolute difference by the standard deviation calculated from measurements of years contributing to the reference period. We calculated the three metrics also for 2011 and contrast anomalies in the 2 years.

We used four reference periods: (1) the entire period when UVI measurements are available (Table 1) up through 2019 (this reference period is different for each site because measurements commenced in different years); (2) the period of (1) but excluding 2011; (3) 2005–2019, the period when OMI data are available (this reference period was included for comparison with anomaly maps shown in Figure 2 derived from OMI data); and (4) the period of (3) excluding 2011.

Both daily and monthly anomalies were computed. For daily anomalies, data measured on a given day in 2020 were compared with data from the same day in previous years from the reference period. For monthly anomalies, data from 2020 were averaged over March, April, May, and June and compared with similar averages from the reference period. Only months with at least 26 days of data were used in the analysis.

Results shown in section 3 are based on noontime UVI measurements. We repeated calculations for the daily maximum UVI and daily erythemal doses and show these results in SI. Anomalies for the three UV quantities are generally similar; noteworthy differences are discussed in sections 3 and 4. We also assembled maps of monthly TOC and UVI anomalies from OMI data for latitudes north of 45°N and compared UVI anomalies extracted from these maps with anomalies calculated from the ground‐based measurements.

## 3 Results

We first present results for daily anomalies, followed by monthly anomalies assessed with ground and satellite data.

### 3.1 Daily TOC and UVI Anomalies

**Figure 1:**
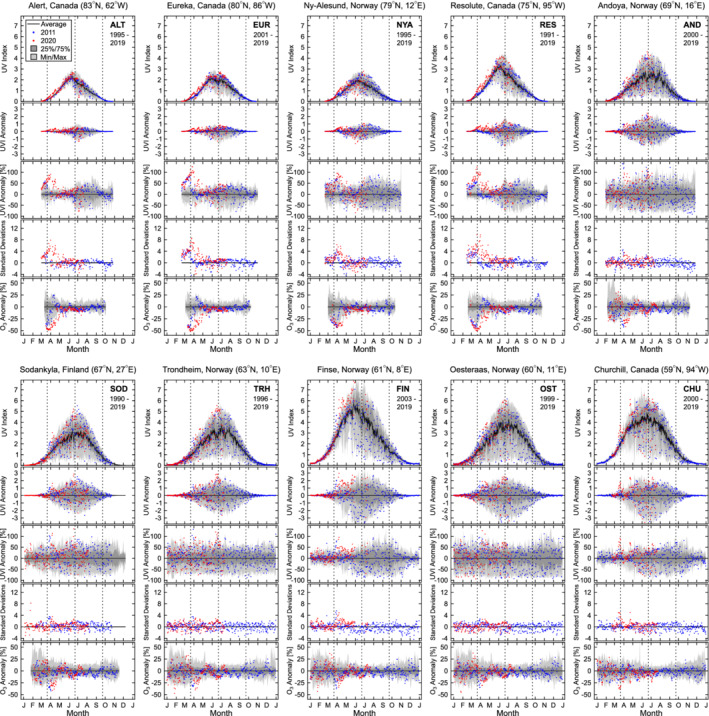
Variation and anomalies of the noontime UVI measured at the 10 sites. The top (first) panel for each site compares noontime UVI measurements performed in 2011 (blue dots) and 2020 (red dots) with the average noontime UVI (black line), the interquartile range (dark shading), and the range of historical minima and maxima (light shading). Average and ranges were calculated from measurements of the range of years indicated in the top right corner of the panel below the site acronym, excluding 2011. The second panel shows 2011 and 2020 UVI anomalies in absolute terms, calculated as the difference between measurements in these years and the climatological mean. The third panel shows relative UVI anomalies calculated as the percentage departure from the climatological mean. The fourth panel indicates the number of standard deviations by which measurements in 2011 and 2020 exceed the climatological mean. The last (fifth) panel shows relative ozone anomalies calculated from satellite measurements. Vertical broken lines in all panels indicate the times of the vernal equinox, summer solstice, and autumnal equinox, respectively.

Figure 1 shows daily UVI and TOC anomalies for 2011 and 2020 relative to reference period (2). In late March 2020, TOCs were up to 50% below the mean for sites in the high Arctic (ALT, EUR, NYA, and RES; see Table 1 for acronyms). For European sites, the largest TOC anomalies occurred in early April with deviations ranging between −30 and −40%. As the polar vortex is continuously changing its shape and position, the location of the low‐ozone episode varies with time. The Canada stations were affected about 1 week earlier than the European sites.

Relative UVI anomalies exceeding 90% can be observed for several days during late March and early April 2020 at all 10 sites. The largest anomaly of 140% occurred at SOD on 6 April 2020. For sites in the Canadian Arctic (ALT, EUR, and RES), anomalies peaked between 17 and 31 March. For European sites above the Arctic Circle (NYA, AND, and SOD), the peak occurred in the first week of April. Relative anomalies for CHU show two distinct peaks, on 18 March and 20 April. European sites below the Arctic Circle (TRH, FIN, and OST) were less affected by the low‐ozone event because the polar vortex did not extend this far South. While anomalies exceeding 100% were also observed on several days at these sites, the effect of clouds on UVR is larger at lower latitudes, and anomalies therefore occur over a background of higher variability.

Most periods of large UVI anomalies coincide with periods of low ozone for all sites. There are, however, also anomalies greater than 100% during mid‐June at AND, SOD, and TRH that cannot be linked in a straightforward manner to the low‐ozone episode during March and April as suggested by Karpechko et al. (2013) for the 2011 low‐ozone episode.

Anomalies exceed 3 SD at all sites but OST. Anomalies exceed 6 SD at ALT, EUR, RES, and NYA and 8 SD at EUR and RES. The largest excess of 9.8 SD was observed at RES on 19 March. These large values exemplify how extraordinary the event in spring 2020 was.

UVR is predominantly controlled by the solar elevation (Weatherhead et al., 2005). Hence, absolute anomalies are generally larger at lower‐latitude sites where the Sun is higher in the sky. Absolute anomalies in March and April for sites north of 70°N remained therefore below 1 UVI unit despite large relative anomalies. With the exception of CHU (UVI = 2.4 on 21 April) and FIN (UVI = 3.0 on 6 April), absolute anomalies were below 2 UVI units at the remaining sites.

At the four sites closest to the North Pole (ALT, EUR, RES, and NYA), TOC and UVI anomalies were on a similar trajectory in 2011 and 2020 up to the spring equinox. TOC returned to normal levels soon afterward in 2011, while TOC remained reduced (and UVI elevated) in 2020 until about 1 April. UVI anomalies quantified by the three metrics were therefore considerably larger in 2020 compared to 2011.

Anomalies calculated from the daily maximum UVI and the daily erythemal dose (SI) are generally slightly smaller than anomalies for the noontime UVIs discussed above. This phenomenon was already noted for the low‐ozone event in 2011 (Bernhard et al., 2013). For the daily dose, the effect can partly be explained by the fact that changes in ozone affect erythemal UVR more strongly when the Sun is higher in the sky (Seckmeyer et al., 2008). Because the solar elevation in the morning and afternoon is smaller than at noon, changes in ozone therefore affect daily doses slightly less than noontime measurements. For example, the maximum relative anomaly at RES is 122% for the noontime UVI and 117% for the daily dose. Smaller anomalies for the daily maximum UVI can be explained by the fact that this quantity is less affected by clouds than measurements averaged over 2 hr. The climatological mean therefore tends to be larger, resulting in smaller anomalies as explained in more detail by Bernhard et al. (2013).

### 3.2 Monthly TOC and UVI Anomalies Derived From OMI Measurements

**Figure 2:**
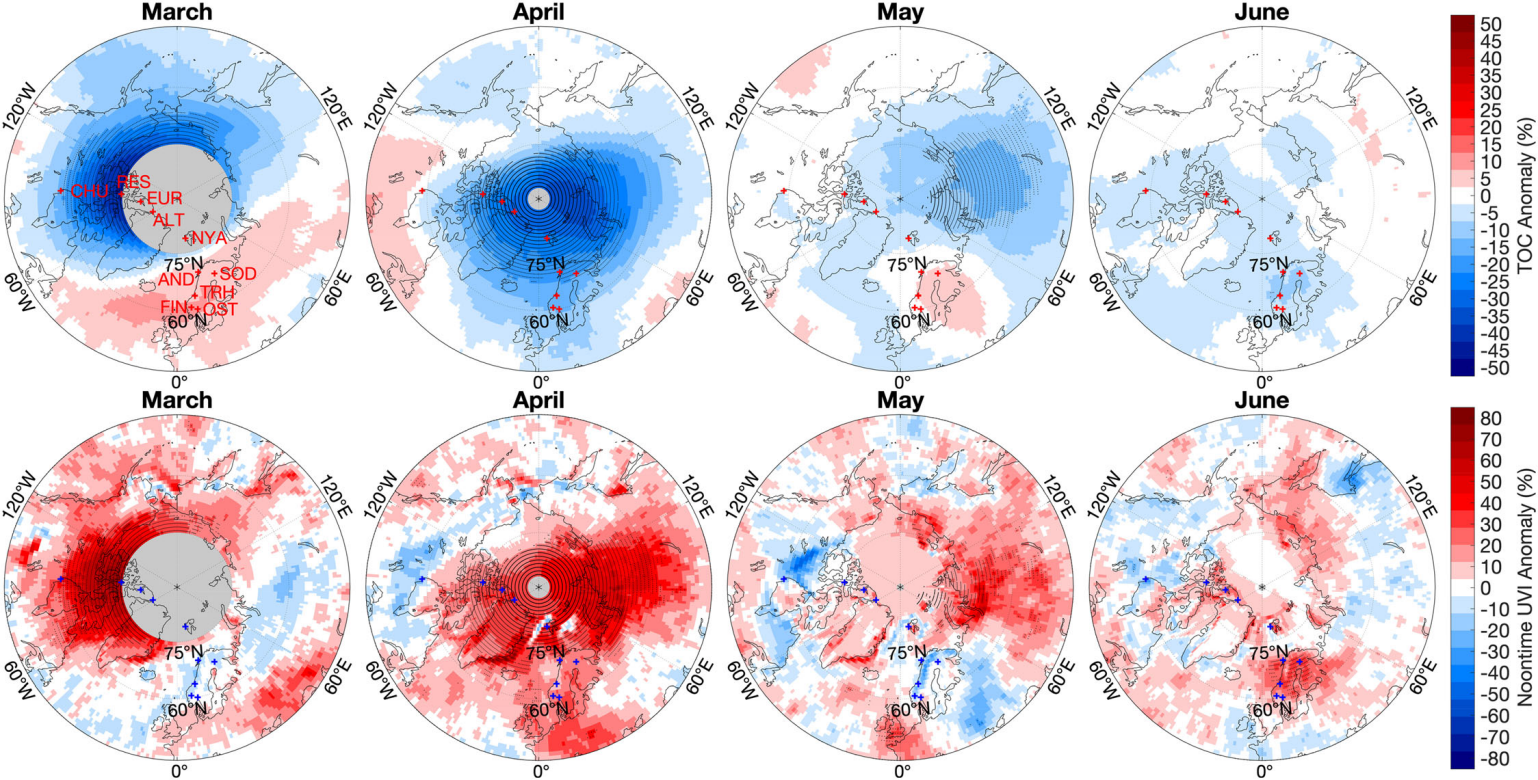
Anomalies of TOC (%) (top) and noontime UVI (%) (bottom) for March, April, May, and June 2020. Anomalies are relative to 2005–19 averages, including the year 2011. Stippling indicates pixels where anomalies exceed 3 SD. Gray‐shaded areas centered at the North Pole in the maps for March and April indicate latitudes where no OMI data are available because of the lack of sunlight at this time of year. Locations of ground stations are indicated by crosses in every map, with labels added to the first map.

Figure 2 shows spatial deviations of monthly average TOCs and UVIs for March, April, May, and June 2020 from the historical (2005–2019) mean estimated from OMI data.

In March, OMI TOC anomalies are largest over Northern Canada; the maximum deviation is −40%. Areas with high UVIs roughly match areas with low TOCs and vice versa, but UVI anomalies have larger spatial variability because of their added dependence on clouds and albedo. Monthly average UVI anomalies over the Canadian Arctic range between 30% and 70%. TOC and UVI anomalies exceed 3 SD over Northern Canada and the Arctic Ocean north of Canada. Note that the low‐ozone area was displaced toward the western hemisphere resulting in average or slightly below average UVIs over the Nordic countries and Siberia.

For April, TOC anomalies are negative for virtually all areas north of 60°N. The maximum anomaly is −35% and is centered over the Arctic Sea north of Siberia. UVI anomalies are positive over a vast area, including Northern Canada, Greenland, Northern Europe, and Siberia. The maximum anomaly is 78% and is north of Siberia. Anomalies exceed 3 SD almost everywhere north of 70°N.

During the breakup of the polar vortex in mid‐to‐late May (Lawrence et al., 2020; Manney et al., 2020), areas below the remains of the polar vortex with abnormally low TOCs still persisted over Siberia. OMI data indicate UVI anomalies of up to 60% but there are no ground‐based instruments in this region to confirm these large departures. TOC anomalies in June were small and exceeded 3 SD only for 4 pixels, west of TRH. TOCs were 5% to 10% below the mean of the area enclosing the 10 ground stations; UVIs were elevated by up to 30% over this region.

### 3.3 Monthly TOC and UVI Anomalies at the Ground Stations

**Figure 3:**
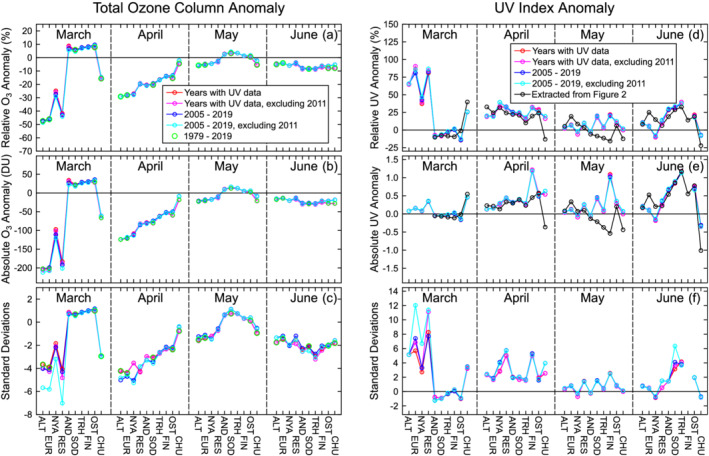
Anomalies of monthly means of TOC (left) and noontime UVI (right) for 2020 as a function of site (sorted with latitude increasing from left to right) and month. Anomalies are quantified as relative differences in percent (a, d), absolute differences (b, e), and multiples of standard deviations (c, f). Anomalies were calculated relative to different reference periods as indicated in the legends. UVI anomalies were derived from ground‐based measurements, except for the data set indicated by the black line, which was extracted from the maps shown in Figure 2. TOC anomalies were calculated from satellite observations. See Table 1 for site acronyms.

Figure 3 shows monthly TOC and UVI anomalies for March, April, May, and June 2020 at the 10 ground stations. These anomalies were computed relative to the four reference periods introduced in section 2.2. TOC departures relative to 1979–2019 (the period of the satellite era) were also assessed. Figure 3 indicates good agreement of results calculated for all reference periods. The good consistency confirms that conclusions drawn from the anomaly maps of Figure 2 (which were based on OMI measurements from 2005 to 2019) are also valid for longer periods. (Note that anomalies referenced to period (4), 2005–2019 excluding 2011, are somewhat larger because this period does not include low‐ozone episodes that have occurred in the past.) The following assessment is based on results referenced to period (1), the period with the longest UVI data record.

For March, TOC anomalies for ALT, EUR, and RES ranged from −42% to −47% and were about 4 SD below the mean. Anomalies at NYA and CHU were −25% and −16%, respectively. Conversely, anomalies at the Norwegian (except NYA) and Finish sites, which were outside the polar vortex in March, exceeded the mean by 6% to 9%. TOC anomalies for April showed a systematic dependence on latitude, changing from −42% at ALT to −4% at CHU. TOC anomalies for May varied between −6% and 3%, while anomalies for June were between −4% and −9%. These deviations are about 2 SD below the mean.

UVI anomalies for March at the Canadian Arctic sites ranged between 65% and 86% and exceeded the mean by 3 to 6 SD. In contrast, the monthly average UVI over the European sites (excluding NYA) was slightly below average. Average UVIs for April were elevated at all sites, ranging from 16% at TRH and CHU to 34% at NYA. With the exception of NYA, AND, and CHU, average UVI anomalies for May were also positive, but anomalies were smaller compared to April and within 2 SD of the mean. Anomalies for June were larger and exceeded 25% at AND, SOD, and TRH; anomalies at SOD and TRH were 3 and 4 SD events, respectively.

As mentioned above, absolute UVI anomalies for March and April remained small (i.e., below 0.6 UVI units except for the anomaly of 1.2 UVI units at FIN) because solar elevations for these months are low.

Figure 3 also shows UVI anomalies calculated from OMI observations, extracted from the maps of Figure 2. With the exception of data for CHU, anomalies calculated from ground and OMI data agree well for March, April, and June. For May, notable differences between the two data sets exist for SOD, TRH and FIN. These discrepancies are likely caused by local conditions as discussed in section 4.

Monthly anomalies calculated from the daily maximum UVI and the daily erythemal dose (see SI) agree within the expected range with noontime anomalies discussed above. Anomaly plots similar to those of Figure 3 for 2011 are also available in SI and confirm that anomalies in 2020 were considerably larger than in 2011. For example, while TOC was depressed by about 45% at ALT, EUR, and RES during March 2020, TOC was lower by only 30% at these sites in 2011. UVI anomalies therefore remained below 35% in 2011, while increases of up to 86% were observed in 2020. Furthermore, the low‐ozone period in 2011 extended only up to 3 April and the effect on UVR was therefore very limited. For instance, monthly UVI anomalies for April 2011 did not exceed 1.7 SD at any site, while anomalies in April 2020 exceeded 1.5 SD at all sites, with a maximum of 5.1 SD.

## 4 Discussion

Monthly anomalies calculated from ground‐based and satellite observations generally agree within a few percent (Figure 3). Exceptions include CHU in April, May, and June and SOD, TRH, and FIN in May. Differences between ground and satellite data at TRH and FIN generally peak in April and May (Bernhard et al., 2015). These discrepancies are likely caused by a mismatch between the albedo climatology used in the satellite retrieval and the actual albedo. Albedo, in turn, is affected by the timing of snowmelt, which was unusually late at SOD and FIN in 2020. In addition, FIN is near a mountain top and the area around the site is not representative of the satellite pixel.

TOC and UVI anomalies in May were within the normal range (i.e., within ±2 SD) at the 10 sites, but a region with anomalies exceeding 3 SD is apparent in satellite data over Siberia (Figure 2). The high UVIs seen at SOD and TRH in June exceed the mean by 4 SD, while TOC is only reduced by 2 SD. Further analysis revealed that unusually nice weather with several cloudless days contributed to the high monthly means for June 2020. Karpechko et al. (2013) provided evidence that the large Arctic ozone loss in the spring of 2011 increased the March–August cumulative erythemal clear‐sky UV dose in the Northern Hemisphere extratropics by 3–4%. A similar study has not been done for the situation in 2020. It is beyond the scope of this paper to assess to what degree the low‐ozone event, associated with chemical destruction in the vortex, early in 2020 may have contributed to the relatively low TOC observed over Scandinavia in June, for example, via dispersal of ozone‐depleted air from the vortex as it broke up during May.

Despite large relative increases in UVR resulting from the low‐ozone event, the noontime UVI observed in March and April has remained below 2.2 UVI units for sites north of 70°N. As the ground and most of the Arctic Ocean are still covered by snow and ice during these months, it is unlikely that the low‐ozone episode had a tangible effect on ecosystems. The largest impact on life and human health may have occurred in June when the UVI was enhanced beyond 3 SD in Norway and Finland, exceeding 5 UVI units for several consecutive days in TRH and OST.

## 5 Conclusions

During March and April 2020, TOC was exceptionally low over the Arctic, resulting in record‐breaking increases in UVR radiation. UVIs observed in 2020 exceeded historical measurements by more than 75% for several days at all sites analyzed. Historical means were surpassed by more than 6 SD at several locations. Monthly mean UVIs were 75% larger than normal in northern Canada for March and elevated by about 25% at all sites for April. Large increases in UVR like those observed in 2020 could reoccur during the next decades in years with a cold and persistent polar vortex as long as concentrations of ozone depleting substances in the atmosphere remain elevated (Wohltmann et al., 2020).

## Supporting information

Supporting Information S1

## Data Availability

The data sets used here are publicly available as follows: OMI Level 3 gridded data used for Figure 2. Ozone: https://disc.gsfc.nasa.gov/datasets/OMTO3e_003/summary website (Bhartia, 2012). UVR: https://disc.gsfc.nasa.gov/datasets/OMUVBd_003/summary website (Hovila et al., 2013). TOMS and OMI overpass files: https://acdisc.gesdisc.eosdis.nasa.gov/data/ [registration required]. UVR data from Canadian sites (https://woudc.org/). UVR data from Norwegian sites (https://github.com/uvnrpa/Minute_Data, https://doi.org/10.5281/zenodo.4043039) (Johnsen et al., 2020). UVR data from Sodankylä (https://litdb.fmi.fi/luo0002_data.php).
