# Supplementary material for: Record‐Breaking Increases in Arctic Solar Ultraviolet Radiation Caused by Exceptionally Large Ozone Depletion in 2020
Source: Geophys Res Lett. 2020 Dec 10;47(24):e2020GL090844. doi: 10.1029/2020GL090844 (PMC9285682; doi:10.1029/2020GL090844)
Supplement: Supplementary file 1 — Supporting Information S1 [file GRL-47-e2020GL090844-s001.pdf]

AGU PUBLICATIONS

Geophysical Research Letters

Supporting Information for

Record-Breaking Increases in Arctic Solar Ultraviolet Radiation Caused by Exceptionally Large Ozone Depletion in 2020

Germar H. Bernhard[1], Vitali E. Fioletov[2], Jens-Uwe Grooß[3], Iolanda Ialongo[4], Bjørn Johnsen[5], Kaisa Lakkala[6], Gloria L. Manney[7,8], Rolf Müller[3], and Tove Svendby[9]

[1]Biospherical Instruments Inc., San Diego, CA, USA; [2]Environment and Climate Change Canada, Toronto, ON, Canada; [3]Forschungszentrum Jülich, Jülich, Germany; [4]Finnish Meteorological Institute, Helsinki, Finland; [5]Norwegian Radiation and Nuclear Safety, Østerås, Norway; [6]Finnish Meteorological Institute, Sodankylä, Finland; [7]NorthWest Research Associates, Socorro, NM, USA; [8]New Mexico Institute of Mining and Technology, Socorro, NM, USA; [9]NILU – Norwegian Institute for Air Research, Kjeller, Norway.

Contents of this file

Figure S1: Variation and anomalies of the **daily noontime UV Index** in 2011 and 2020
Figure S2: Variation and anomalies of the **daily maximum UV Index** in 2011 and 2020
Figure S3: Variation and anomalies of the **daily erythemal UV dose** in 2011 and 2020
Figure S4: Anomalies of the monthly mean of the **noontime UV Index for 2011**
Figure S5: Anomalies of the monthly mean of the **noontime UV Index for 2020**
Figure S6: Anomalies of the monthly mean of the **daily maximum UV Index for 2020**
Figure S7: Anomalies of the monthly mean of the **daily erythemal UV dose for 2020**

Introduction

Figures 1 of the main paper shows variation and anomalies of the noontime UV Index measured at the 10 sites discussed in the main paper. Figures S2 and S3 shown below provide similar graphics for the daily maximum UV Index (Figure S2) and the daily erythemal UV dose (Figure S3). Figure S1 is identical to Figure 1 of the main paper and was included here to ease comparison between the various datasets.

Figure 3 of the main paper shows anomalies of monthly means of the total ozone column and the noontime UV Index for 2020. Figures S6 and S7 shown below provide similar graphics for monthly means of the daily maximum UV Index (Figure S6) and the daily erythemal UV dose (Figure S7). Figure S5 is identical to Figure 3 of the main paper and was included here to ease comparison between the various datasets. In addition, Figure S4 shows monthly anomalies for 2011 based on the noontime UV Index. By toggling between Figures S4 and S5, anomalies in 2011 and 2020 can be compared.

Noontime UV Index; all years with UV data

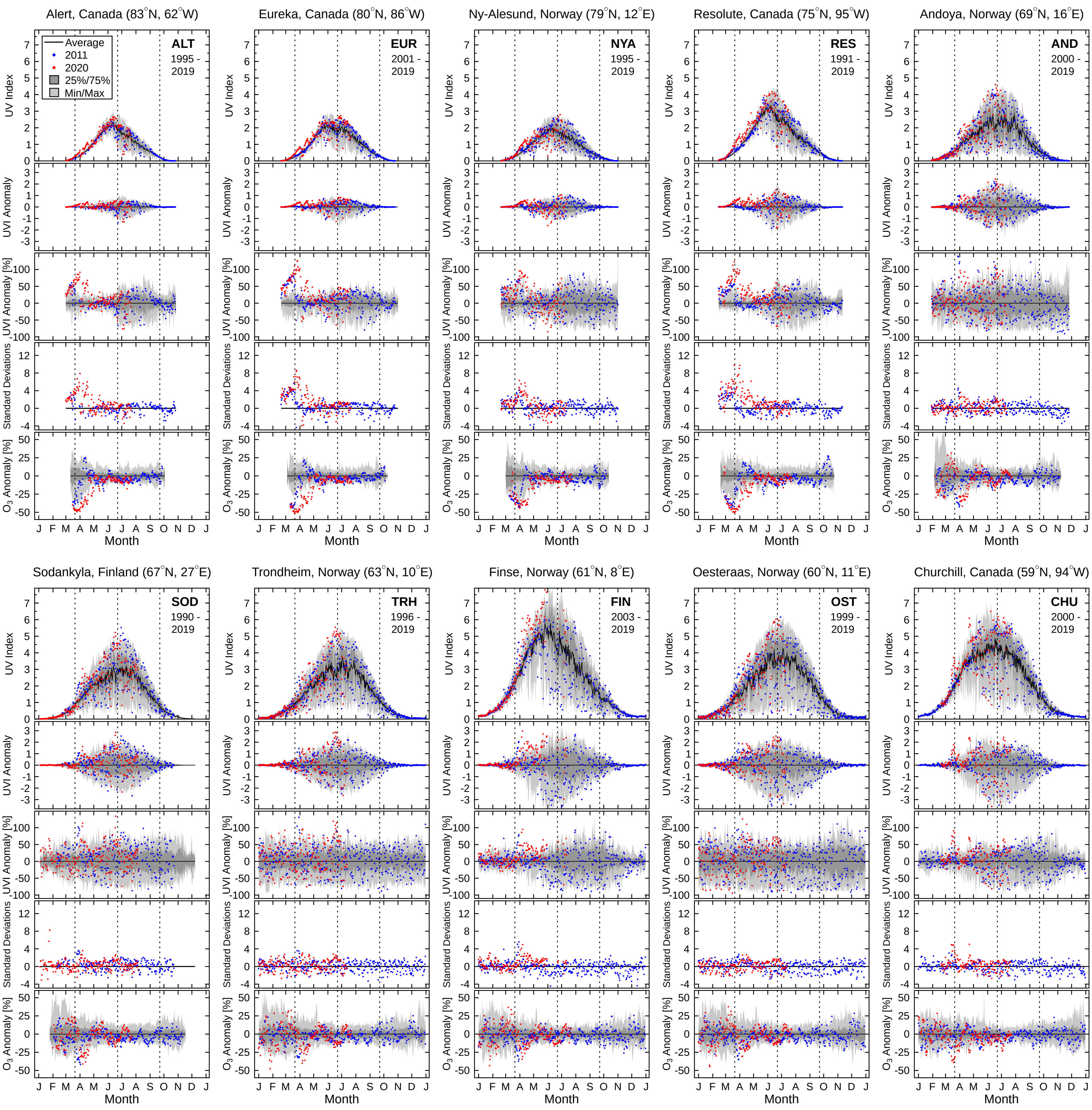

Figure S1. Variation and anomalies of the **noontime UVI** at the 10 sites. The top (first) panel for each site compares noontime UVI measurements performed in 2011 (blue dots) and 2020 (red dots) with the average noontime UVI (black line), the interquartile range (dark shading), and the range of historical minima and maxima (light shading). Average and ranges were calculated from measurements of the range of years indicated in the top-right corner of the panel below the site acronym, excluding 2011. The second panel shows 2011 and 2020 UVI anomalies in absolute terms, calculated as the difference between measurements in these years and the climatological mean. The third panel shows relative UVI anomalies calculated as the percentage departure from the climatological mean. The fourth panel indicates the number of standard deviations by which measurements in 2011 and 2020 exceed the climatological mean. The last (fifth) panel shows relative ozone anomalies calculated from satellite measurements. Vertical broken lines in all panels indicate the times of the vernal equinox, summer solstice, and autumnal equinox, respectively.

Daily maximum UV Index; all years with UV data

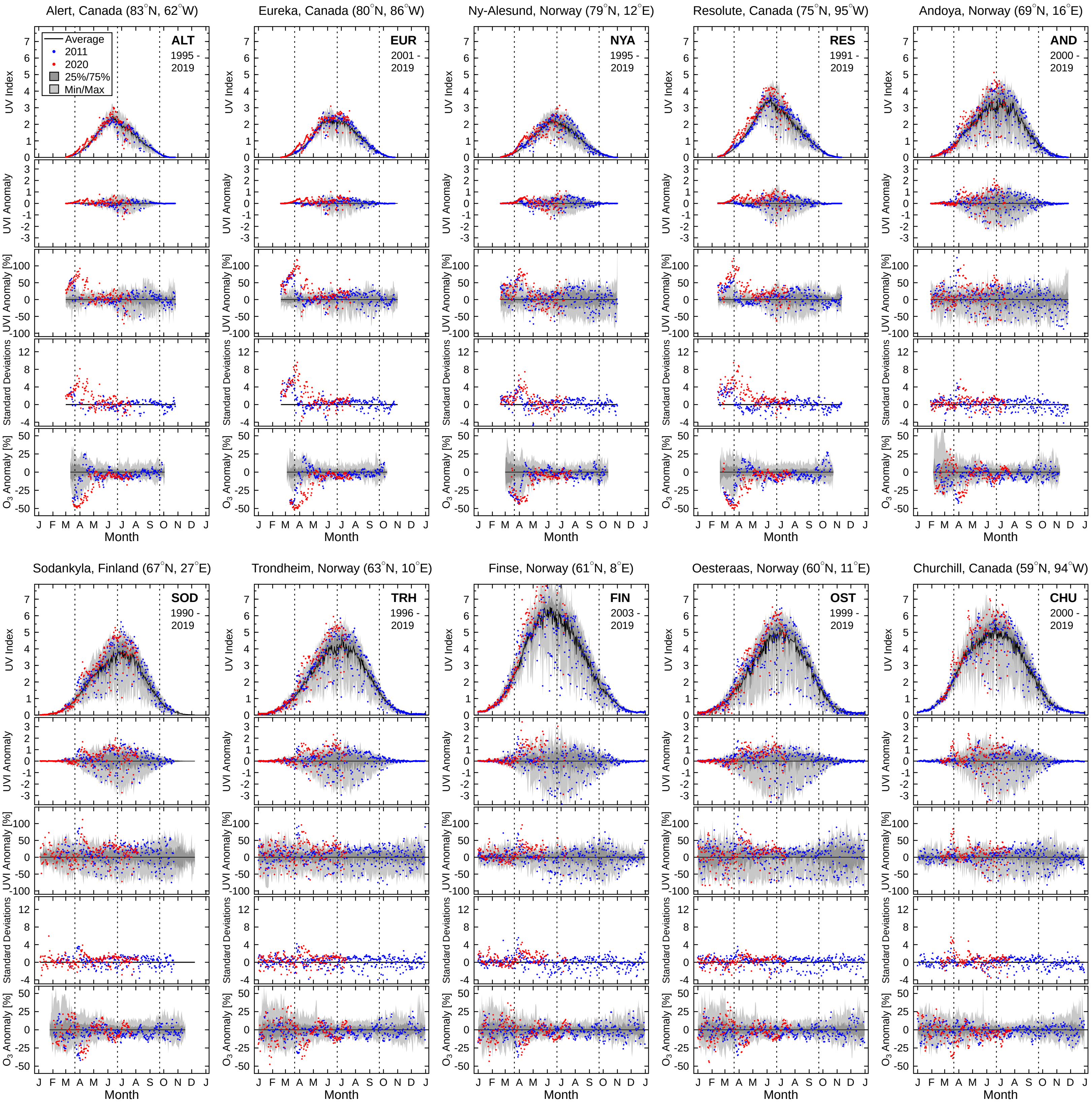

Figure S2. Variation and anomalies of the **daily maximum UV Index** at the 10 sites. The top (first) panel for each site compares measurements of the daily maximum UV Index performed in 2011 (blue dots) and 2020 (red dots) with the average noontime UVI (black line), the interquartile range (dark shading), and the range of historical minima and maxima (light shading). Average and ranges were calculated from measurements of the range of years indicated in the top-right corner of the panel below the site acronym, excluding 2011. The second panel shows 2011 and 2020 UVI anomalies in absolute terms, calculated as the difference between measurements in these years and the climatological mean. The third panel shows relative UVI anomalies calculated as the percentage departure from the climatological mean. The fourth panel indicates the number of standard deviations by which measurements in 2011 and 2020 exceed the climatological mean. The last (fifth) panel shows relative ozone anomalies calculated from satellite measurements. Vertical broken lines in all panels indicate the times of the vernal equinox, summer solstice, and autumnal equinox, respectively.

Daily erythemal UV dose; all years with UV data

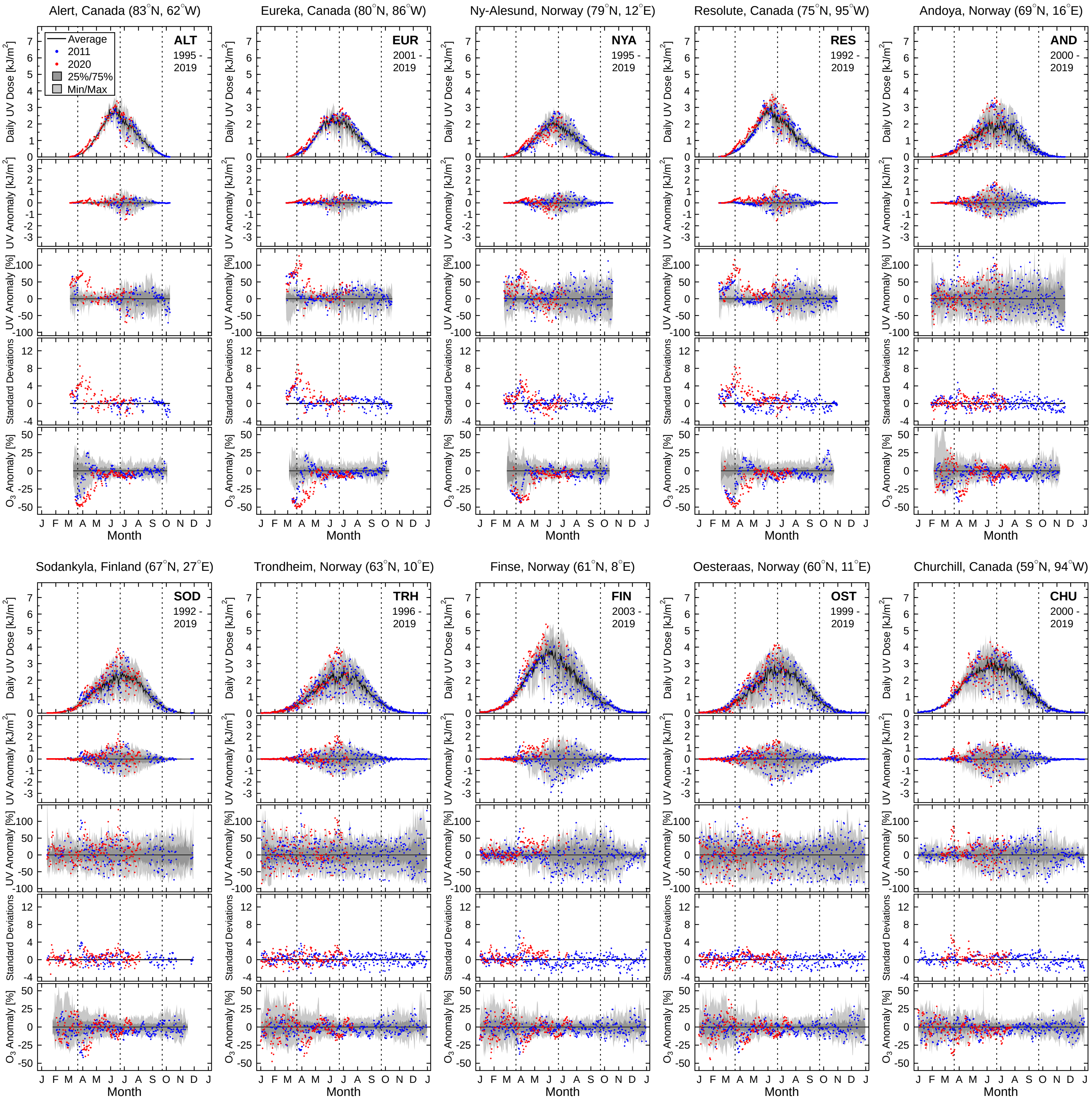

Figure S3. Variation and anomalies of the **daily erythemal UV dose** at the 10 sites. The top (first) panel for each site compares measurements of the daily erythemal UV dose performed in 2011 (blue dots) and 2020 (red dots) with the average noontime UVI (black line), the interquartile range (dark shading), and the range of historical minima and maxima (light shading). Average and ranges were calculated from measurements of the range of years indicated in the top-right corner of the panel below the site acronym, excluding 2011. The second panel shows 2011 and 2020 UVI anomalies in absolute terms, calculated as the difference between measurements in these years and the climatological mean. The third panel shows relative UVI anomalies calculated as the percentage departure from the climatological mean. The fourth panel indicates the number of standard deviations by which measurements in 2011 and 2020 exceed the climatological mean. The last (fifth) panel shows relative ozone anomalies calculated from satellite measurements. Vertical broken lines in all panels indicate the times of the vernal equinox, summer solstice, and autumnal equinox, respectively.

Noontime UV Index; anomaly for 2011

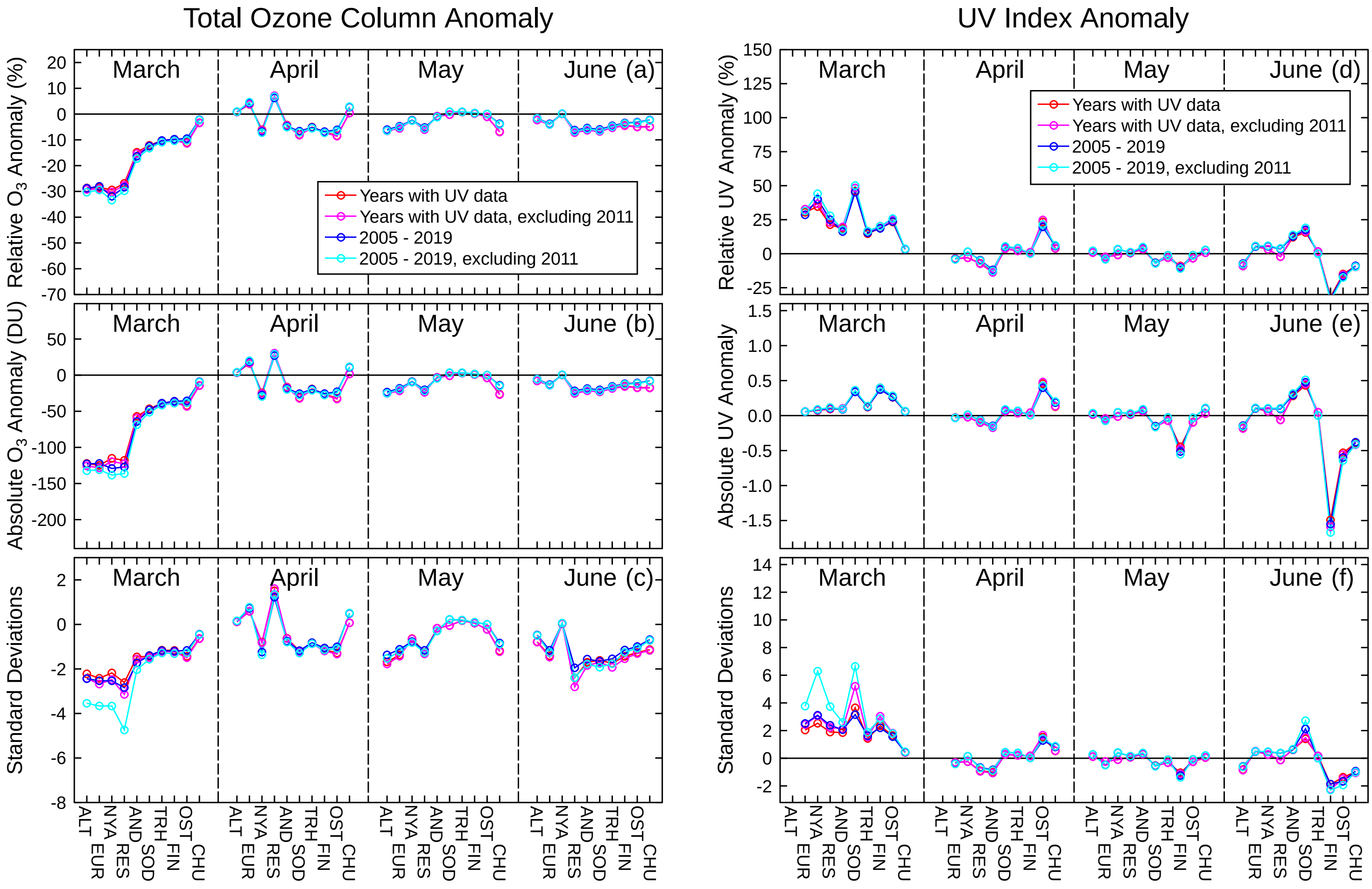

Figure S4. Anomalies of monthly means of TOC (left) and **noontime UVI** (right) **for 2011** as a function of site (sorted with latitude increasing from left to right) and month. Anomalies are quantified as relative differences in percent (a, d), absolute differences (b, e), and multiples of standard deviations (c, d). Anomalies were calculated relative to different reference periods as indicated in the legends. UVI anomalies were derived from ground-based measurements. TOC anomalies were calculated from satellite observations. See Table 1 for site acronyms.

Noontime UV Index; anomaly for 2020

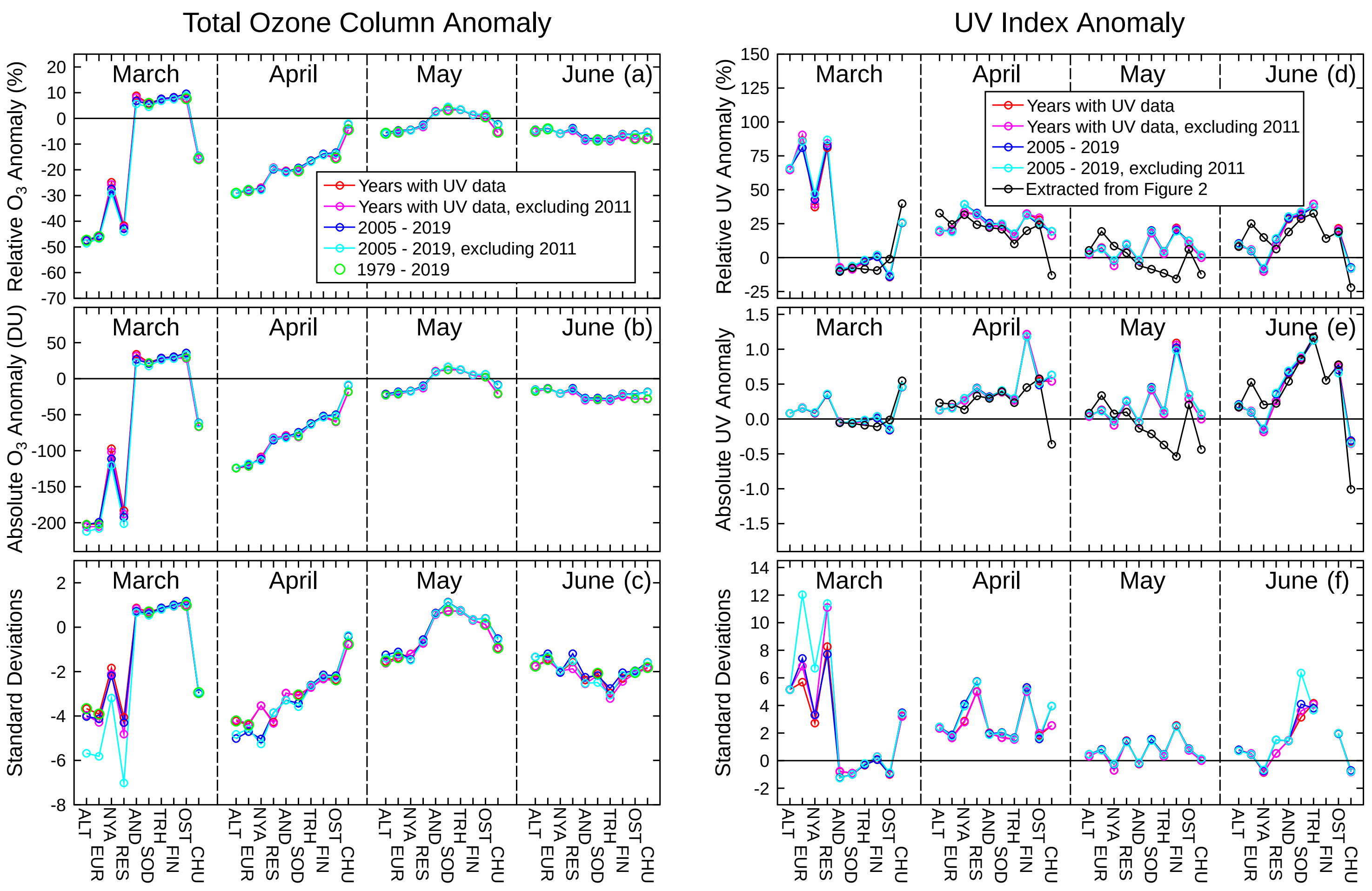

Figure S5. Anomalies of monthly means of TOC (left) and **noontime UVI** (right) **for 2020** as a function of site (sorted with latitude increasing from left to right) and month. Anomalies are quantified as relative differences in percent (a, d), absolute differences (b, e), and multiples of standard deviations (c, d). Anomalies were calculated relative to different reference periods as indicated in the legends. UVI anomalies were derived from ground-based measurements, except for the dataset indicated by the black line, which was extracted from the maps shown in Figure 2. TOC anomalies were calculated from satellite observations. See Table 1 for site acronyms.

Daily Maximum UV Index; anomaly for 2020

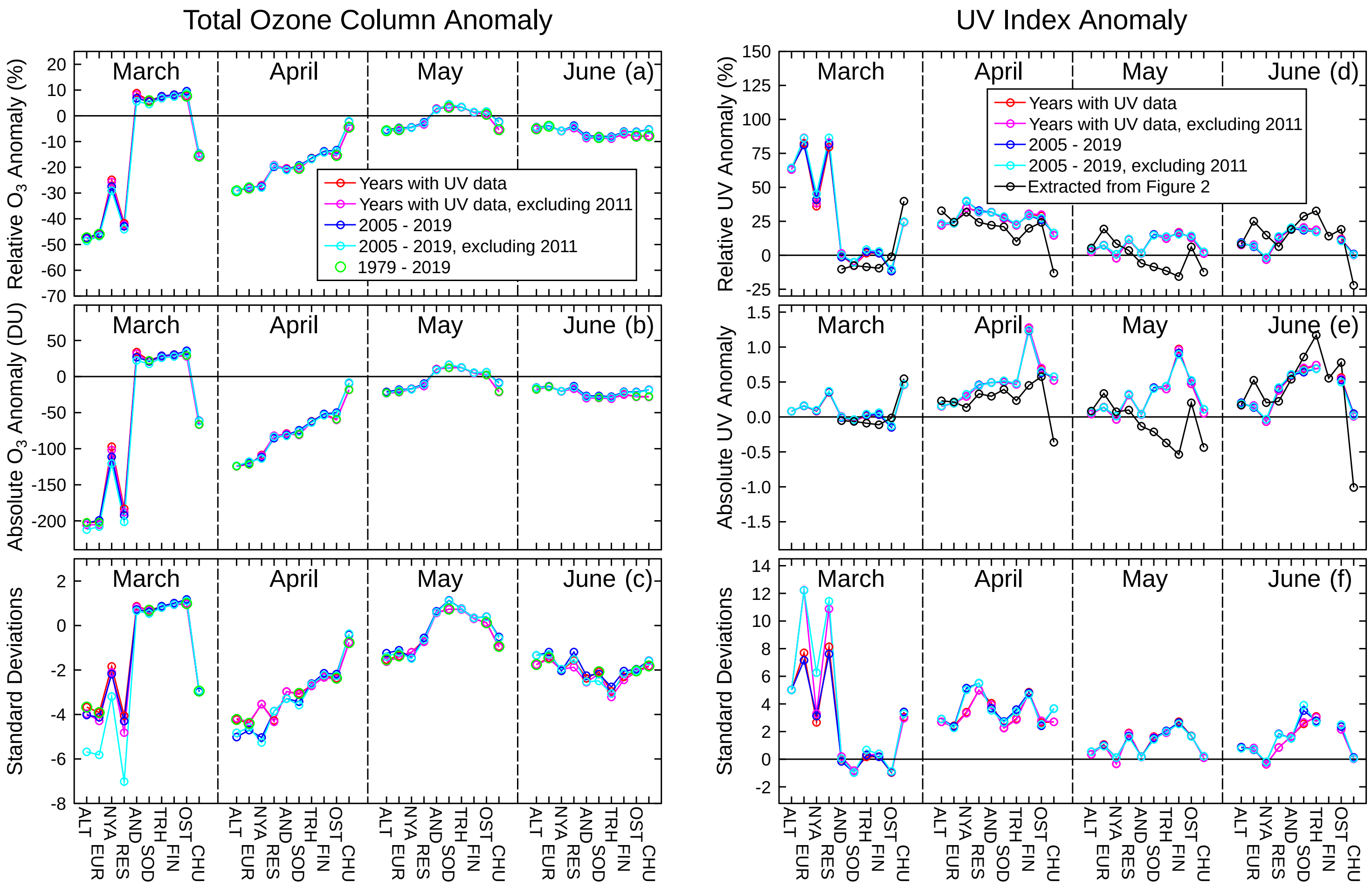

Figure S6. Anomalies of monthly means of TOC (left) and the **daily maximum UV Index** (right) **for 2020** as a function of site (sorted with latitude increasing from left to right) and month. Anomalies are quantified as relative differences in percent (a, d), absolute differences (b, e), and multiples of standard deviations (c, d). Anomalies were calculated relative to different reference periods as indicated in the legends. UVI anomalies were derived from ground-based measurements, except for the dataset indicated by the black line, which was extracted from the maps shown in Figure 2. TOC anomalies were calculated from satellite observations. See Table 1 for site acronyms.

Daily Erythemal UV Dose; anomaly for 2020

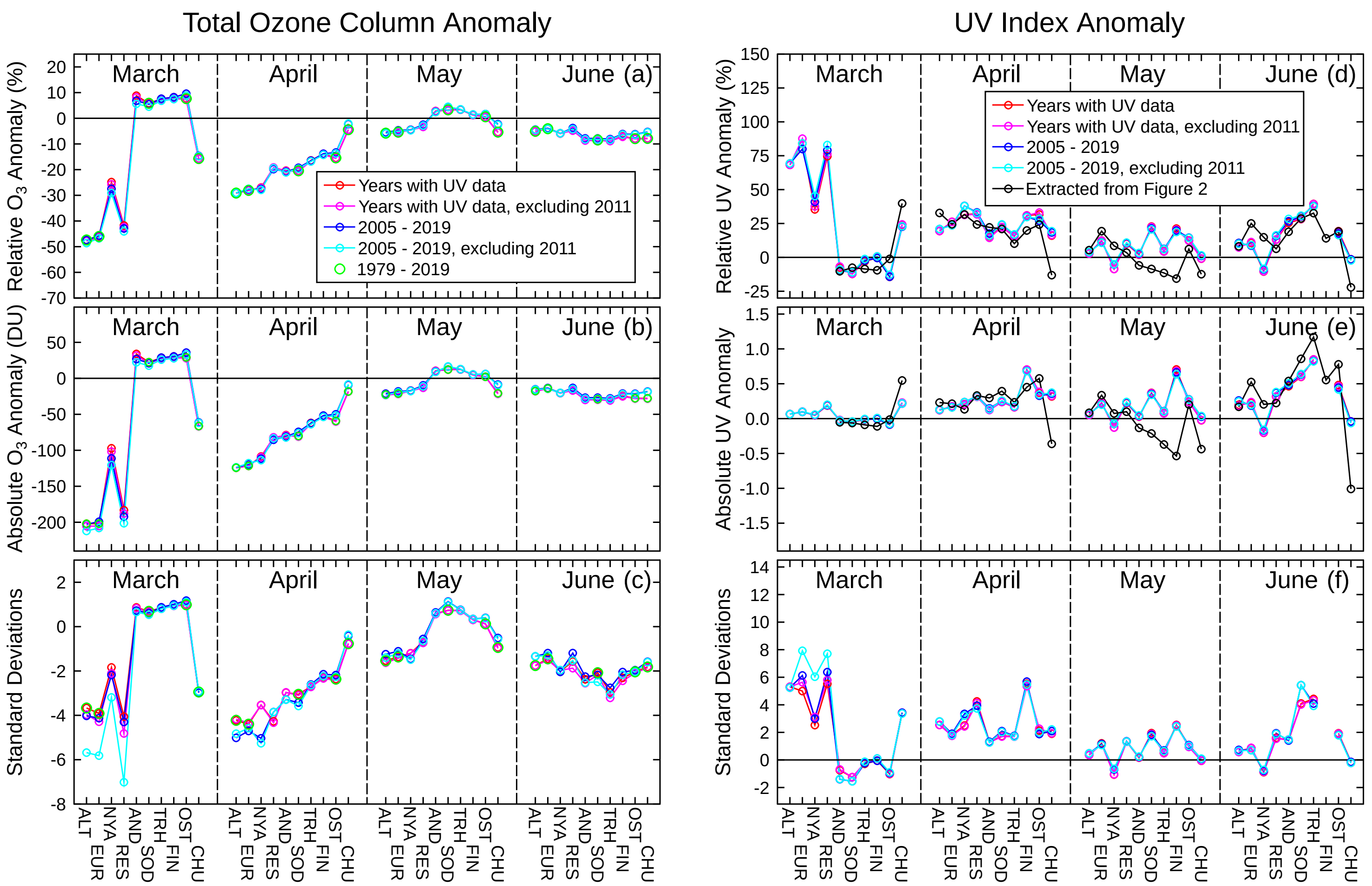

Figure S7. Anomalies of monthly means of TOC (left) and the **daily erythemal UV dose** (right) **for 2020** as a function of site (sorted with latitude increasing from left to right) and month. Anomalies are quantified as relative differences in percent (a, d), absolute differences (b, e), and multiples of standard deviations (c, d). Anomalies were calculated relative to different reference periods as indicated in the legends. UVI anomalies were derived from ground-based measurements, except for the dataset indicated by the black line, which was extracted from the maps shown in Figure 2. TOC anomalies were calculated from satellite observations. See Table 1 for site acronyms.
